# Modeling Radiotherapy Induced Normal Tissue Complications: An Overview beyond Phenomenological Models

**DOI:** 10.1155/2016/2796186

**Published:** 2016-12-01

**Authors:** Marco D'Andrea, Marcello Benassi, Lidia Strigari

**Affiliations:** Laboratory of Medical Physics and Expert Systems, Regina Elena National Cancer Institute, Rome, Italy

## Abstract

An overview of radiotherapy (RT) induced normal tissue complication probability (NTCP) models is presented. NTCP models based on empirical and mechanistic approaches that describe a specific radiation induced late effect proposed over time for conventional RT are reviewed with particular emphasis on their basic assumptions and related mathematical translation and their weak and strong points.

## 1. Introduction

Modern radiotherapy techniques allow unprecedented levels of accuracy, precision, and conformity in target localization, patient setup, and dose delivery thanks to the aid of many different imaging modalities.

Contemporary treatment strategies almost always involve delivering higher doses to the targeted tissue with the aim of improving tumor control, but before such approaches can be safely implemented an accurate and reliable knowledge on toxic effects on surrounding tissues has to be secured.

With the aim of normal tissue preservation many models have been proposed to describe radiation induced complications mostly focusing on late complications which, being irreversible, are considered to have the highest impact on the patient quality of life.

In this, as in most overviews [1–5], normal tissue complication probability (NTCP) models have been divided into mechanistic and (semi)empirical, according to the level of detail in tissue structure that is introduced. Ideally models should be able to accommodate the body of knowledge coming from cellular radiobiology and more specifically the linear quadratic (LQ) model of cell kill and its more sophisticated evolutions that incorporate cell proliferation, cycle effects, and repair as well as local environmental effects and vascularization. The mechanistic models which almost invariably rely on the paradigm on viewing the tissue as a cooperative collection of functional subunits that allow preservation of the tissue functionalities are able to integrate the LQ model in a more straightforward way.

This overview focuses on the description of tissue organization without any initial assumption on the subunit response to radiation. Our approach stresses the mathematical translation of the features of the presented models to better expose their versatility and the opportunities for further developments. Indeed, radiobiological modeling needs a quite complex mathematical toolbox, mirroring the complexity of the biological systems. Each organ is not just an agglomerate of cells but it has an underlying architecture/organization that is the very basis of its functional role, enabling many different strategies (renewal/replacement of damaged cells, intricate microscopic repair pathways, etc.) to successfully deal with radiation damage [6].

Many of these radiobiological models have been integrated into Treatment Planning Systems [7–10] sometimes in a simplified form to allow a biological optimization of the dose delivery or a ranking of competing treatment strategies. This can be seen as a mandatory first step towards a patient specific design of a radiotherapy care path.

## 2. The Lyman-Kutcher-Burman Model

### 2.1. The Pure Lyman Model

The most widely used NTCP model in clinical radiobiology is the Lyman model [11].

The core of the model is a fit of frequency data collected for chosen clinical toxicity endpoints and for a particular class of dose irradiation patterns, namely, those patterns where a given portion of an organ or tissue volume absorbs a spatially uniform dose and the rest of its volume absorbs (ideally) no dose at all [12]. If we label as *S*_*D*_ the point set within the irradiated organ where the dose takes the constant dose value *D*, the chosen fitting function is assumed to be given by the following expression [13]:

(1) F=121+erf⁡t2;t=D−D50oe·μSD−noemoe·D50oe·μSD−noe=D·μSDnoe−D50oemoe·D50oe,

where *μ*(*S*_*D*_) is the measure of the support set *S*_*D*_ (assuming the set *O*, representing the whole organ, to have measure 1); *D*_50__*o*_^*e*^, *n*_*o*_^*e*^, and *m*_*o*_^*e*^ are fitting parameters, specific for organ *o* and endpoint *e*, and erf⁡(*x*) is the error function, defined as

(2) $$\begin{array}{c} \mathrm{erf}\left(\;x\;\right)≝\frac{2}{\sqrt{\pi }}\overset{x}{\underset{0}{\int }}e^{-\xi^{2}}d\xi . \end{array}$$

As the only features of the dose map that are included in the model are its constant value and the measure of its support, it is clear that the actual position and shape of the set *S*_*D*_ within the organ are considered irrelevant.

In more technical terms, if we define a random indicator $Y_{D\left(\underline{r}\right)}^{o,e}$ such that $Y_{D\left(\underline{r}\right)}^{o,e}=1$ flags the event that endpoint *e* is observed in organ *o* when the latter absorbs the dose $D\left(\underline{r}\right)$, we can say that at this stage we assume to know the probability $P\left(Y_{D\cdot {𝕀}_{S_{D}}\left(\underline{r}\right)}^{o,e}=1|n_{o}^{e},m_{o}^{e},{D_{50}}_{o}^{e}\right)$ only for dose maps $D\left(\underline{r}\right)$ of the form $D\cdot {𝕀}_{S_{D}}\left(\underline{r}\right)$ for any *S*_*D*_, where $\underline{r}$ is a point in the organ under investigation and ${𝕀}_{S_{D}}\left(\underline{r}\right)$ is the indicator function of the set *S*_*D*_; that is,

(3) $$\begin{array}{c} I_{S_{D}}\left(\;\underline{r}\;\right)=\left\{\begin{array}{cc} 1 & \forall \underline{r}\in Ο:{\underline{r}\in S}_{D}\\ 0 & \forall \underline{r}\in Ο:\underline{r}\notin S_{D}. \end{array}\right. \end{array}$$

This knowledge is summarized by a database of triples (*n*_*o*_^*e*^, *m*_*o*_^*e*^, *D*_50__*o*_^*e*^).

### 2.2. The Kutcher-Burman Interpolation

Having described the radiobiological consequences of the partial uniform dose distributions, the model needs to be extended to arbitrary distributions; that is, we want to know the probability $P\left(Y_{D\left(\underline{r}\right)}^{o,e}=1|n_{o}^{e},m_{o}^{e},{D_{50}}_{o}^{e}\right)$ for arbitrary $D\left(\underline{r}\right)$. We are thus facing an interpolation/extrapolation problem in the domain of dose maps and the missing information must be fed in, through some further assumption. Many solutions have been proposed to this problem [14, 15] the most widely accepted being the so-called “Kutcher-Burman histogram reduction scheme” [16–20]. This interpolation algorithm relies on the concept of Dose Volume Histogram (DVH) [21, 22], which we now briefly introduce. First of all, to be more adherent to the clinical practice and to avoid dimensional issues, we note that a dose map $D\left(\underline{r}\right)$ is usually given in the form $D\cdot h\left(\underline{r}\right)$ where *D* is the prescription dose and $h\left(\underline{r}\right)$ is a dimensionless function (the “normalized dose distribution”).

The range of $h\left(\underline{r}\right)$ can be divided into, say, *N* intervals (bins) of width Δ, each interval defining its inverse image. To a chosen binning we can associate the set of couples {(*h*_*i*_, *μ*(*S*_*h*_*i*__))} where one representative value, *h*_*i*_ (e.g., the lower boundary or the middle point), in each dose interval is matched to the size *μ* of the inverse image, *S*_*h*_*i*__, of that interval. This set of couples is called differential (normalized) DVH (dDVH).

The key idea of the Kutcher-Burman interpolation is to consider each couple (*h*_*i*_, *μ*(*S*_*h*_*i*__)) of the dDVH under investigation as describing an independent partial uniform distribution $D\cdot h_{i}\cdot {𝕀}_{S_{h_{i}}}\left(\underline{r}\right)$ to which a new, equivalent (here “equivalent” means being mapped to the same real number by *ℱ*), partial uniform distribution, $D\cdot {𝕀}_{T_{h_{i}}}\left(\underline{r}\right)$, is associated. This new equivalent partial uniform distributions is taken to have a support *T*_*h*_*i*__ whose size is fixed by the requirement of preserving the complication probability, that is to say, the value of *t* in (1). In formulas we have

(4) $$\begin{array}{c} \frac{D\cdot h_{i}-{D_{50}}_{o}^{e}\cdot {\left(\mu \left(S_{h_{i}}\right)\right)}^{-n_{o}^{e}}}{{m_{o}^{e}\cdot {D_{50}}_{o}^{e}\cdot \left(\mu \left(S_{h_{i}}\right)\right)}^{-n_{o}^{e}}}=\frac{D-{D_{50}}_{o}^{e}{\cdot \left(\mu \left(T_{h_{i}}\right)\right)}^{-n_{o}^{e}}}{{m_{o}^{e}\cdot {D_{50}}_{o}^{e}\cdot \left(\mu \left(T_{h_{i}}\right)\right)}^{-n_{o}^{e}}} \end{array}$$

from which we derive

(5) $$\begin{array}{c} \mu \left(\;T_{h_{i}}\;\right)={h_{i}}^{1/n_{o}^{e}}\mu \left(\;S_{h_{i}}\;\right). \end{array}$$

The final assumptions are as follows: (a) consider the supports *T*_*h*_*i*__ as disjoint (which we can do in accordance with the assumption that the actual position of the dose absorbing regions is irrelevant) so that the partial uniform distribution $D\cdot {𝕀}_{{⋃}_{i}T_{h_{i}}}\left(\underline{r}\right)$ can be defined and (b) take the initial distribution as being equivalent to the latter partial uniform distribution for which we are allowed to use formula (1) to calculate a value of *t* and a complication probability. Indeed,

(6) $$\begin{array}{c} \mu \left(\;\underset{i}{⋃}T_{h_{i}}\;\right)=\underset{i}{\sum }\mu \left(\;T_{h_{i}}\;\right)=\underset{i}{\sum }{h_{i}}^{1/n_{o}^{e}}\mu \left(\;S_{h_{i}}\;\right). \end{array}$$

We have that

(7) tD−D50oe·μ⋃iThi−noemoe·D50oe·μ⋃iThi−noe=D−D50oe·∑ihi1/noeμShi−noemoe·D50oe·∑ihi1/noeμShi−noe.

A further trivial manipulation of the last formula yields

(8) $$\begin{array}{c} t=\frac{D\cdot {\left(\sum_{i}{h_{i}}^{1/n_{o}^{e}}\mu \left(S_{h_{i}}\right)\right)}^{n_{o}^{e}}-{D_{50}}_{o}^{e}}{m_{o}^{e}\cdot {D_{50}}_{o}^{e}}. \end{array}$$

And it is clear that the product in the numerator can be interpreted as a uniform dose delivered to the whole organ volume which has the same complication probability of the original distribution; hence it is named Equivalent Uniform Dose (EUD):

(9) $$\begin{array}{c} EUD≝D\cdot {\left(\;\sum_{i}{h_{i}}^{1/n_{o}^{e}}\mu \left(\;S_{h_{i}}\;\right)\;\right)}^{n_{o}^{e}}. \end{array}$$

It is easily checked that a change in the normalization for $h\left(\underline{r}\right)$ does not change the value of *t*, provided that the prescription dose is scaled accordingly (indeed formula (8) is most often written as *t* = (*D*_ref_ · [∑_*i*_(*D*_*i*_/*D*_ref_)^1/*n*_*o*_^*e*^^ *μ*(*S*_*D*_*i*__)]^*n*_*o*_^*e*^^ − *D*_50__*o*_^*e*^)/(*m*_*o*_^*e*^ · *D*_50__*o*_^*e*^) for arbitrary *D*_ref_; however note that (*D*_ref_ · [∑_*i*_(*D*_*i*_/*D*_ref_)^1/*n*_*o*_^*e*^^ *μ*(*S*_*D*_*i*__)]^*n*_*o*_^*e*^^ − *D*_50__*o*_^*e*^)/(*m*_*o*_^*e*^ · *D*_50__*o*_^*e*^) = ((*D*/*D*) · *D*_ref_ · [∑_*i*_(*D*_*i*_/*D*_ref_)^1/*n*_*o*_^*e*^^ *μ*(*S*_*D*_*i*__)]^*n*_*o*_^*e*^^ − *D*_50__*o*_^*e*^)/(*m*_*o*_^*e*^ · *D*_50__*o*_^*e*^) = (*D* · [∑_*i*_(*D*_*i*_/((*D*/*D*_ref_) · *D*_ref_))^1/*n*_*o*_^*e*^^ *μ*(*S*_*D*_*i*__)]^*n*_*o*_^*e*^^ − *D*_50__*o*_^*e*^)/(*m*_*o*_^*e*^ · *D*_50__*o*_^*e*^) = (*D* · (∑_*i*_ *h*_*i*_^1/*n*_*o*_^*e*^^ *μ*(*S*_*h*_*i*__))^*n*_*o*_^*e*^^ − *D*_50__*o*_^*e*^)/(*m*_*o*_^*e*^ · *D*_50__*o*_^*e*^)).

Note also that in the above reasoning the values *n*_*o*_^*e*^, *m*_*o*_^*e*^, *D*_50__*o*_^*e*^ are not dependent on the dose map; therefore they are expected to be those listed in the “elementary” database. Continually updated versions of this body of knowledge can be found in the literature [23], most notably the QUANTEC collaboration [24] which is the reference for daily clinical practice in radiotherapy.

### 2.3. Issues on Fractionation Effects

When the Lyman model was proposed, patient irradiation had a simple ballistician that delivered dose distributions with sharp penumbras. As a consequence the basic assumption that dose delivery was performed on a dichotomic ‎basis, with irradiated tissue absorbing all the dose at the intended fractionation and the rest of the tissues absorbing no dose at all, was fairly accurate. In a few years, however, technological advances allowed irradiations strategies that, in spite of a much better performance in concentrating the high dose region around the tumour tissue, could induce highly nonuniform low dose deposit in surrounding healthy tissues. This motivated, at least partially, the introduction of the Kutcher-Burman reduction scheme, which then needed to accommodate also the fact that different portions of the irradiated volume receive the accumulated dose in different fraction sizes according to their position in the inhomogeneous dose map. The most used approach to solve this issue is a nonlinear rescaling of the dose axis according to the BED formula of the LQ model [25, 26]. This solution is obtained by forcing microscopic radiobiology into a model that has no built-in slot to account for “microscopic resolution.” However, once this step is accepted, additional microscopic details coming from dedicated in vitro experiments can be included such as incomplete repair, track structure effects [27, 28], and high dose linearization of survival (i.e., the LQL model) [29, 30].

## 3. The Models Based on Functional Subunits

### 3.1. The Threshold Poisson-Binomial (TPB) Models: Critical Volume Model and Critical Element Model

The Critical Volume Model [31–33] relies on the identification of an “atomic” biological entity, usually denoted as Functional Subunit (FSU), whose response to ionizing radiation can be described as a Bernoulli trial, with an associated dichotomic random variable (random indicator) *X*, taking the value 1 with probability *p* if the FSU has been “inactivated,” that is, it is unable to perform its biological task after the irradiation, and the value 0 with probability 1 − *p* if the FSU is still functioning properly.

It is also assumed that a definite collection of *N* of these atomic entities forms a tissue, that is, a macroscopic, composite, biological entity, whose status after irradiation is evaluated from the observation of some chosen biological endpoint.

It is only from this viewpoint that the FSUs are considered as “atomic” in the sense that all the information about their interaction with ionizing radiation is summarized by the inactivation probability *p*. In fact, to arrive at a consistent expression relating absorbed dose to inactivation probability the internal structure of the FSUs must be considered.

If we define *X*_*i*_ as the random indicator associated with the *i*th FSU, then the sum *K* = ∑_*i*_^*N*^ *X*_*i*_ is a random variable which counts the number of FSUs that have been inactivated by radiation.

In the standard formulation of the model, the FSUs are assumed independent, and the behavior of their macroscopic aggregate, that is, the tissue, is described by the variable *K* just introduced, together with a characteristic threshold *t*_*o*_^*e*^. These two can be combined in a new, global, random indicator *Y*_*o*_^*e*^ = *H*(*K* − *t*_*o*_^*e*^), where *H* is the Heaviside step function. *K* and *t*_*o*_^*e*^ are related in such a way that if more than *t*_*o*_^*e*^ FSUs are inactivated by radiation, that is, *K* ≥ *t*_*o*_^*e*^, then the tissue *o*, as a whole, becomes unable to perform one or more of its functions and its impaired status, *Y*_*o*_^*e*^ = 1, is flagged by the biological endpoint *e* under observation.

From the assumed independence of the FSUs it follows that if *p*_*i*_ is the probability associated with the variable *X*_*i*_ taking the value 1, then the variable *K* has a probability distribution, frequently named “Poisson-binomial,” which reduces to the usual binomial distribution if all the *p*_*i*_s are identical. In the following, an arbitrary distribution of inactivation probabilities among the *N* FSUs, that is, the *N*-tuple of probabilities (*p*_1_, *p*_2_,…, *p*_*N*_), will be denoted as $\vec{p}$, a uniform probability distribution will be denoted as $p{\vec{1}}_{N}$, where ${\vec{1}}_{N}$ is the indicator *N*-tuple (1,1,…, 1), and a partial uniform probability distribution will be denoted as $p{\vec{1}}_{M}$, where ${\vec{1}}_{M}$ is the indicator *N*-tuple (1,…, 1,0,…, 0) having the first *M* components equal to 1 and the remaining *N* − *M* equal to 0.

Given $\vec{p}$, the probability $P\left(K=k|\vec{p}\right)$ that exactly *k* out of *N* FSUs are inactivated, that is, the Poisson-binomial probability mass function, is

(10) PK=k ∣ p→fNk,p→=∑AkN∏i∈AkNpi∏j∉AkN1−pj,

where the summation ranges over all the possible subcollections *𝒜*_*k*_^*N*^ of *k* out of *N* FSUs and, therefore, has $\left(\begin{array}{c} N\\ k \end{array}\right)$ terms.

From expression (10) it is clear that a permutation of the indices of the components of the vector $\vec{p}$ leaves the value of $P\left(K=k|\vec{p}\right)$ unchanged. Since the index *i* locates a particular FSU in space, this reflects the fact that independence of the FSUs wipes out any global spatial feature of the tissue under investigation.

A different expression is often used for (1) by first summing over the FSUs that share the same value of inactivation probability [34, 35]. The components of the vector $\vec{p}$ are therefore clustered in, say, *r* probability bins, with *r* ≤ *N*, each bin *h* being referred to *n*_*h*_ FSUs having the common inactivation probability value *p*_*h*_ and, of course, ∑_*h*=1_^*r*^ *n*_*h*_ = *N*. Expression (10) can thus be rewritten as

(11) fNk,p→=∑j1,j2,…,jr∑h=1rjh=k ∏h=1rnhjhphjh1−phnh−jh,

that is, as a constrained summation of products of binomial probability mass functions, each describing the inactivation of *j*_*h*_ out of *n*_*h*_ FSUs in the *h*th probability bin. From (11) we see that if each bin refers to exactly one FSU, that is, *r* = *N* and *n*_*h*_ = 1  ∀*h*, we obtain again expression (10); if, on the other hand, there is just one bin, that is, we have a uniform probability distribution, then we obtain the usual binomial probability mass function:

(12) $$\begin{array}{c} f_{N}\left(\;k,p{\vec{1}}_{N}\;\right)=\left(\begin{array}{c} N\\ k \end{array}\right)p^{k}{\left(\;1-p\;\right)}^{N-k}. \end{array}$$

A closed form expression for (11) can be found, for example, in [34]. Discrete Fourier Transform techniques can be used to obtain a closed form also for the general expression (10) [36].

From (10) or (11) it can be shown that [37]

(13) EK ∣ p→∑h=1rnhph=Np−VarK ∣ p→∑h=1rnhph1−ph=EK ∣ p→−∑h=1rnhph2=Np−−p2−.

With the usual notation $\overset{-}{z}=\left(1/N\right)\sum_{j=1}^{N}z_{j}$ for the sample mean of an *N*-tuple (*z*_1_, *z*_2_,…, *z*_*N*_). Introducing the (biased) sample variance $\sigma_{p}^{2}=\left(1/N\right)\sum_{j=1}^{N}\left(p_{j}^{2}-\overset{-}{p}\right)=\overset{-}{p^{2}}-{\overset{-}{p}}^{2}$ it follows from (11) that

(14) $$\begin{array}{c} Var\left(\;K\;|\;\vec{p}\;\right)=Var\left(\;K\;|\;\overset{-}{p}{\vec{1}}_{N}\;\right)-N\sigma_{p}^{2} \end{array}$$

which shows how the variance of *S* increases with increasing uniformity among the inactivation probabilities and attains its maximum value when all the *p*_*i*_s are identical.

The probability $P\left(K<l|\vec{p}\right)$ that at most *l* − 1 FSUs are inactivated defines the cumulative distribution function:

(15) $$\begin{array}{c} P\left(\;K<l\;|\;\vec{p}\;\right)≝F_{N}\left(\;l,\vec{p}\;\right)=\overset{l-1}{\underset{k=0}{\sum }}f_{N}\left(\;k,\vec{p}\;\right). \end{array}$$

The complement to 1 of this function gives the probability that at least *l* FSUs are inactivated:

(16) $$\begin{array}{c} P\left(\;K\geq l\;|\;\vec{p}\;\right)=1-F_{N}\left(\;L,\vec{p}\;\right). \end{array}$$

When *l* is equal to the tissue characteristic threshold *t*_*o*_^*e*^, we are looking at the event *Y*_*o*_^*e*^ = 1 that we identify with the loss of some functionality in the tissue, which shows up in the observation of the related biological endpoint, and the corresponding probability could be called “tissue failure probability” (TFP).

The customary interpretation of the described probability distributions and the related nomenclature is such that when 1 ≤ *t*_*o*_^*e*^ < *N* we are actually looking at healthy tissue or an organ being damaged by ionizing radiation, the probabilities *p*_*l*_ can span the whole range [0,1], and the probability $P\left(K\geq t_{o}^{e}|\vec{p}\right)$ is usually called NTCP. The lower extreme, *t*_*o*_^*e*^ = 1, describes a tissue where damage to a single FSU is enough to trigger the endpoint. This scenario actually has its own model, namely, the so-called Critical Element model, which, although being a special case of the Critical Volume, was introduced somewhat before the latter [38–41]. At the upper extreme (*t*_*o*_^*e*^ = *N*), instead, we are looking at a tumour undergoing therapeutic irradiation, the probabilities *p*_*l*_ are, hopefully, all close to 1, and the probability $P\left(K\geq N|\vec{p}\right)$ is called “Tumour Control Probability” (TCP).

The number of FSUs is usually very large and approximate expressions for (15) that are easier to handle can be used, the normal and the Poisson one being the most popular. The normal approximation stems from the well-known DeMoivre-Laplace limit theorem generalized to the Poisson-binomial distribution. Indeed, if the vector $\vec{p}$ is such that $Var\left(K|\vec{p}\right)\geq 100$ [34] we have that

(17) PK≥toe ∣ p→~1−12πVarK ∣ p→∫0toee−z−EK ∣ p→2/2VarK ∣ p→dz,

where $E\left(K|\vec{p}\right)$ and $Var\left(K|\vec{p}\right)$ are given by formulas (13). On the other hand, for the case of therapeutic irradiation of a tumour since *p*_*h*_ = 1 − *s*_*h*_, with *s*_*h*_ ≪ 1, the variable *S* = *N* − *K* which counts the number of FSUs still active has approximately a Poisson distribution whose zero class identifies the event of total FSU inactivation; that is,

(18) $$\begin{array}{c} P\left(\;K\geq N\;|\;\vec{p}\;\right)=P\left(\;S\leq 0\;|\;\vec{s}\;\right)=e^{-E\left(S\;|\;\vec{s}\right)}, \end{array}$$

where $\vec{s}={\vec{1}}_{N}-\vec{p}$ and $E\left(S|\vec{s}\right)=\sum_{h=1}^{r}n_{h}q_{h}$.

It can be shown [42–44] that $f_{N}\left(k,\vec{p}\right)$ in (10) is well approximated also by the following:

(19) fNk,p⋆1→M=Mkp⋆k1−p⋆M−k,0≤k≤M0,M<k≤N

which is a Poisson-binomial probability mass function that, from the viewpoint of representation (11), has only *r* = 2 bins: *p*_1_ = *p*_⋆_, *n*_1_ = *M*, *p*_2_ = 0, and *n*_2_ = *N* − *M*.

*M* and *p*_⋆_ are defined by the system of equations

(20) Mp⋆=EK ∣ p→Mp⋆1−p⋆=VarK ∣ p→,

whose solution, taking into account (13) and the fact that *M* must be an integer, is

(21) p⋆EK ∣ p→−VarK ∣ p→EK ∣ p→=∑h=1rnhph2∑h=1rnhphME2K ∣ p→EK ∣ p→−VarK ∣ p→+12=∑h=1rnhph2∑h=1rnhph2+12

with ⌊ ⌋ being the floor function which outputs the integer part of a real number. Using the sample mean $\overset{-}{p}$ and the sample variance *σ*_*p*_^2^ equations (21) can be rewritten as

(22) p⋆=p2−p−=p−1+σpp−2

(23) MN=1NNp−2p2−+12~11+σp/p−2.

This approximation also works for the cumulative distribution (15) for any *l* and therefore, in particular, for *l* = *t*_*o*_^*e*^. Since *M*/*N* is less than 1, a notion of “effective fraction,” *μ*≝*M*/*N*, of irradiated FSUs comes naturally into play and its value is fixed by the inactivation probabilities according to formula (22). The “equivalent uniform probability,” *p*_⋆_, over the effective fraction of irradiated FSUs can be used to define an Equivalent Uniform Dose via the specification of how the inactivation probability is related to dose, provided that appropriate uniformity assumptions are introduced, as will be explained in the following.

If we introduce the random variable Λ≝*K*/*N* describing the relative amount of inactivated FSUs together with the relative threshold *τ*_*o*_^*e*^≝*t*_*o*_^*e*^/*N* the approximation (17) becomes

(24) PΛ≥τoe ∣ p→~1−12πVarΛ ∣ p→∫0τoee−λ−EΛ ∣ p→2/2VarΛ ∣ p→dλ.

$E\left(K|\vec{p}\right)=NE\left(\Lambda |\vec{p}\right)$ and $Var\left(K|\vec{p}\right)=N^{2}Var\left(\Lambda |\vec{p}\right)$. Using (17) we have

(25) PΛ≥τoe ∣ p→~PΛ≥τoe ∣ p⋆1→M~1−12πμp⋆1−p⋆/N·∫0τoee−λ−μp⋆2/2μp⋆1−p⋆/Ndλ=1−1π·∫−N/2μp⋆/μp⋆1−p⋆N/2τoe−μp⋆/μp⋆1−p⋆e−ξ2dξ.

This formula can be rewritten using the error function erf⁡(*x*) defined as

(26) $$\begin{array}{c} \mathrm{erf}\left(\;x\;\right)≝\frac{2}{\sqrt{\pi }}\overset{x}{\underset{0}{\int }}e^{-\xi^{2}}d\xi \end{array}$$

and the result is

(27) PΛ≥τoe ∣ p→~1−12erf⁡N2τoe−μp⋆μp⋆1−p⋆−erf⁡−N2μp⋆μp⋆1−p⋆.

A number *N* of FSUs large enough to enforce the normal approximation (17) turn the Gaussian integrand in (25) into a *δ*-like distribution and we see that

(28) PΛ≥τoe ∣ p→1−∫0τoeδλ−μp⋆dλ=1−∫−∞∞Hλ−τoeδλ−μp⋆dλ=Hμp⋆−τoe;

therefore tissue failure takes place (with probability one) whenever

(29) $$\begin{array}{c} \mu p_{⋆}\geq \tau_{o}^{e}; \end{array}$$

that is,

(30) $$\begin{array}{c} \overset{-}{p}\geq \tau_{o}^{e}. \end{array}$$

In other words the law of large numbers further erases the details of the distribution of the *p*_*i*_s and the only remaining feature is the arithmetic mean $\overset{-}{p}$.

Even if no specification other than monotonic increase is made about the functional dependence of the FSU inactivation probability on radiation dose, it is clear that, upon choice of a given tissue, that is, a value of *τ*_*o*_^*e*^, formula (28) predicts a sharp transition in $P\left(\Lambda \geq \tau_{o}^{e}|\vec{p}\right)$ as a function of dose which is not observed in clinical data. This could be amended by going back to formula (25)–(27), but the fact that *N* is sufficiently large to strongly concentrate the Gaussian density function around its expectation value seems hardly questionable.

To explain the gentler slope of $P\left(\Lambda \geq \tau_{o}^{e}|\vec{p}\right)$ it is necessary to recognize the sampling character of the clinical toxicity frequency data that we are investigating. Indeed, choosing a tissue does not specify a value of *τ*_*o*_^*e*^, but only its distribution among the population of patients. Given the symmetrical roles of *τ*_*o*_^*e*^ and $\mu p_{⋆}=\overset{-}{p}$ in (28), the same idea works also when a distribution of radiobiological parameters is assumed among the patients so that a given radiation dose pattern, $\vec{D}$, does not yield a definite value of $\vec{p}$ and, therefore, of $\overset{-}{p}$ but, again, a distribution of values. It may be worth pointing out that heterogeneity of radiobiological parameters among the FSUs of a single patient is already taken into account by the assumed heterogeneity of the *p*_*i*_s and it cannot prevent the sharp transition when the number of FSUs becomes very large.

In formulas, if we rewrite (28) as

(31) PYoe=1 ∣ τoe,p→~∬−∞∞δx−p−Hx−yδy−τoedx dy,

where the notation $P\left(Y_{o}^{e}=1|\tau_{o}^{e},\vec{p}\right)$ has now been used in replacement of $P\left(\Lambda \geq \tau_{o}^{e}|\vec{p}\right)$ to parallel the treatment of the LKB model, the above reasoning amounts to substitute *δ*(*y* − *τ*_*o*_^*e*^) with a density distribution *φ*_*o*_^*e*^(·) for the relative threshold and $\delta \left(x-\overset{-}{p}\right)$ with a (dose pattern dependent) density distribution $\psi_{\vec{D}}\left(\cdot \right)$ for $\overset{-}{p}$. The resulting general expression, therefore, becomes

(32) PYoe=1 ∣ φoe·,D→~∬−∞∞ψD→xHx−yφoeydx dy.

Going back one step by leaving $\psi_{\vec{D}}\left(x\right)=\delta \left(x-\overset{-}{p}\right)$ and focusing only on the distribution *φ*_*o*_^*e*^(·) the above formula turns into

(33) $$\begin{array}{c} P\left(\;Y_{o}^{e}=1\;|\;\varphi_{o}^{e}\left(\;\cdot \;\right),\vec{p}\;\right)\sim \overset{1}{\underset{0}{\int }}\varphi_{o}^{e}\left(\;y\;\right)H\left(\;\overset{-}{p}-y\;\right)dy. \end{array}$$

If we denote by Φ_*o*_^*e*^(*y*) the cumulative distribution of *τ* and integrate (31) by parts, we obtain

(34) PYoe=1 ∣ φoe·,p→Hp−−yΦoey01+∫01Φoeyδy−p−dy=Φoep−.

Therefore, varying the radiation dose pattern actually samples the cumulative distribution of the relative threshold *τ*_*o*_^*e*^ through the mean value of the inactivation probability or, in simpler words, the probability of undergoing a tissue failure, given $\vec{p}$, is just the probability of having a relative threshold smaller than $\overset{-}{p}$.

### 3.2. FSU Inactivation Probability

Having shown the assumptions that regulate the organizational response of tissue in the Critical Volume Model and discussed their consequences we must now specify the dose dependence of the FSU inactivation probabilities *p*_*i*_.

The simplest approach, which however somehow spoils the mechanistic attitude of the CV model, is to assume a generic sigmoid shaped dose response curve whose parameters may or may not depend on the position of the FSU within the organ. In the former case we have some means to introduce a spatial feature within the organ, so that the position and shape of the irradiated volume become relevant.

A more mechanistically oriented assumption is the replication of the Critical Volume approach at a smaller scale: the FSU is considered as an aggregate of *N*′ independent stem cells and FSU inactivation occurs when a sufficiently large fraction, *t*′, of these stem cells has been killed by radiation [45, 46]. To complete the picture we can invoke the LQ model to describe the cell killing dependence on radiation dose and we may assume that even a single surviving stem cell can allow the FSU to regenerate; that is, that *t*′ = *N*′:

(35) $$\begin{array}{c} p_{i}={\left(\;1-e^{-\alpha_{i}D_{i}-\beta_{i}D_{i}^{2}}\;\right)}^{N^{\prime }}, \end{array}$$

where the subscript *i*, which, as already pointed out, locates the FSU in space, has been attached also to the radiobiological parameters to allow for their position dependence within the organ.

### 3.3. Nesting TPBs: Kallman's Model

The idea of nesting one Poisson-binomial probability distribution into another allows modeling increasing complexity of tissue organization.

At the level of complexity immediately next to the Critical Volume Model, an organ is viewed as a bundle of *N*_1_ “meta-FSUs” that can be damaged according to the CV model with a *t*_1_ threshold, and each meta-FSU is itself made of *N*_2_ FSUs and again follows the CV model with a *t*_2_ threshold.

Single FSU inactivation can be described, for example, by expression (35).

In formulas for the *h*th meta-FSU, borrowing from (10), (15), and (16), this translates into

(36) $$\begin{array}{c} P_{h}=1-\overset{t_{2}-1}{\underset{i=0}{\sum }}\underset{A_{i}^{N_{2}}}{\sum }\left(\;\underset{j\in A_{i}^{N_{2}}}{\prod }p_{hj}\;\right)\left(\;\underset{l\notin A_{i}^{N_{2}}}{\prod }\left(\;1-p_{hl}\;\right)\;\right). \end{array}$$

And for the whole organ under investigation

(37) PK≥t1 ∣ p→=1−∑k=0t1−1∑AkN1∏m∈AkN1Pm∏n∉AkN11−Pn,

where $\vec{p}$ is the double-indexed vector *p*_*hl*_ of FSU inactivation probabilities and *𝒜*_*i*_^*N*_2_^ and *𝒜*_*k*_^*N*_1_^ are the subcollections of *i* FSUs out of *N*_2_ and *k* meta-FSUs out of *N*_1_, respectively. This general formula is usually restricted to a pure parallel behavior of the organ with respect to the meta-FSUs, [47, 48], that is, *t*_1_ = *N*_1_, and a pure serial behavior of each meta-FSU, that is, *t*_2_ = 1. Therefore we have

(38) $$\begin{array}{c} TFP=\overset{N_{1}}{\underset{m=1}{\prod }}P_{m}=\overset{N_{1}}{\underset{m=1}{\prod }}\left[\;1-\overset{N_{2}}{\underset{j=1}{\prod }}\left(\;1-p_{mj}\;\right)\;\right]. \end{array}$$

As it can be seen by direct inspection of formulas (36)-(37) or (38) there is no invariance for arbitrary index permutation, which means that this model has some built-in global spatial feature. Indeed even if no spatial dependence is assumed for the parameters that regulate FSU response to radiation dose, different positions and shapes of the irradiated volume with respect to the meta-FSU/FSU arrangement yield different values of NTCP. This means that to compare the model to clinical data one needs to take into account spatial details which are most often not available; therefore a simplified version of the model with forced position invariance is used. Such a model can be inspired by (38) after a few observations. First of all, when the inactivation probabilities are assumed identical among the subunits, formula (38) turns into

(39) $$\begin{array}{c} TFP={\left[\;1-{\left(\;1-p\;\right)}^{N_{2}}\;\right]}^{N_{1}}. \end{array}$$

Which can be trivially solved for *p*:

(40) $$\begin{array}{c} p=1-{\left(\;1-{TFP}^{1/N_{1}}\;\right)}^{1/N_{2}}. \end{array}$$

Now we focus on a subset of *a* · *N*_1_ meta-FSUs, each one limited to only *b* · *N*_2_ FSUs and, by analogy with (39), we assume that the failure probability of this subset of FSUs is

(41) TFPa·b1−1−pb·N2a·N1=1−1−TFP1/N1ba·N1=1−1−TFP1/N1a·bN1.

Having used (40) in the second step. This “tile” of tissue with relative volume *a* · *b* = *v* is now by definition our new elementary FSU and we introduce a new parameter termed the “relative seriality” *s*:

(42) $$\begin{array}{c} s=\frac{N_{2}}{N_{2}\cdot N_{1}}=\frac{1}{N_{1}}, \end{array}$$

which is the ratio of the number of the “old” FSUs contained in a meta-FSU over the total number of “old” FSUs. With these new definitions formula (40) can be rewritten as

(43) $$\begin{array}{c} {p=\left[1-{\left(1-{TFP}^{s}\right)}^{v}\right]}^{1/s} \end{array}$$

and allowing the *p*s to have spatial dependence, for an organ comprising *N* “tiles,” we have

(44) TFP1−∏i=1N1−pisv1/s=1−∏i=1N1−TFPisv1/s,

where, as before, *i* labels a position within the organ under investigation. In summary we got rid of the difficulties of formula (38) at the expense of collapsing the tissue complexity into an elementary “tile” which has become the new FSU. The usual binning of the inactivation probabilities yields

(45) $$\begin{array}{c} TFP={\left[\;1-\prod_{h=1}^{r}{\left(\;1-{{TFP}_{h}}^{s}\;\right)}^{v\cdot n_{h}}\;\right]}^{1/s}, \end{array}$$

where *v* · *n*_*h*_ is the relative size of the *h*th dose bin.

## 4. Conclusions

The main aim of the LKB model is to accommodate the available clinical data into a reasonably manageable function, in order to help the clinician in assessing the odds of safe and successful treatment. As long as the clinical needs are involved, any understanding of the underlying biological phenomena is pursued only as long as it is instrumental to the above purpose. From the viewpoint of detailed radiobiological modeling, however, this model can be taken as a summary of the available clinical knowledge.

Quite general features of a normal tissue complication probability model which are already present in the LKB model are the following.

The observation of endpoint *e* in organ *o* is an event that can be encoded in an indexed family of random indicators $Y_{D\left(\underline{r}\right)}^{o,e}:\Omega \to \mathbb{R}$, each described by a Bernoulli distribution. The set *𝔇* of dose maps (*D* ∈ *𝔇*)⇔(*D* : *Ο* → *ℝ*) is the index set for this family.

The only parameter of the Bernoulli distribution, that is, the probability of occurrence of the event, is given by a functional defined on *𝔇*. In particular in the LKB model the functional is of the form

(46) $$\begin{array}{c} \left(\;f\circ {h\circ g}^{-1}\circ \overset{}{\underset{Ο}{\int }}d^{3}\underline{r}g\circ \;\right) \end{array}$$

with *g*(*x*) = *x* ^1/*n*_*o*_^*e*^^, *h*(*y*) = (*y* − *D*_50__*o*_^*e*^)/(*m*_*o*_^*e*^ · *D*_50__*o*_^*e*^) and *f*(*z*) = erf⁡(*z*). Noteworthy is the composition $t\left(y\right)=\left(g^{-1}\circ \int_{Ο}^{}d^{3}\underline{r}g\circ \right)\left(y\right)$, that is, the “histogram reduction,” which is responsible for the dramatic reduction of the dimensionality of the problem.

At this stage, then, a rather featureless sample space is enough to describe experimental data. The TPB models add some details to the sample space *Ω* and related probability structure. The cost of trying to magnify the microscopic biological features is that the experimental noise, that is, the conditions in which data are gathered, has to be taken into account too, and at the same level of detail.

The mechanistic models, here exemplified by the TPB models, have an intrinsic ability to include microscopic biological features of the radiation induced damage; however the parameters needed to describe such details have not been determined for most treatment sites and thus cannot be used in daily clinical practice. Accurate determination of fine structure parameters in a clinical setting is quite a formidable task, even when foreseeable biasing is taken into account in the model framework. In addition, even the QUANTEC parameter database for the widely used LKB model suffers from indeterminacies due to nonuniformity of volume definitions (e.g., hollow organs) and heterogeneity in the quality of data and in the radiobiological assumptions

All these models and the proposed parameters summarizing the features of a specific tissue/organ have originated in the years of two-dimensional and three-dimensional (conformal) radiotherapy. Nevertheless they currently are the only available approaches to predict the expected toxicities deriving from the modern intensity modulated delivery techniques such as IMRT and VMAT.
